# Cefepime Combined with Late-Generation β-Lactamase Inhibitors: Mechanisms of Action, In Vitro Activity, PK/PD Characteristics, Clinical Evidence and Resistance Mechanisms

**DOI:** 10.3390/antibiotics15030263

**Published:** 2026-03-03

**Authors:** Sara Comini, Matteo Boattini, Paolo Gaibani, Gabriele Bianco

**Affiliations:** 1 Operative Unit of Clinical Pathology, Carlo Urbani Hospital, 60035 Ancona, Italy; cominisara@gmail.com; 2 Microbiology and Virology Unit, University Hospital Città della Salute e della Scienza di Torino, 10126 Turin, Italy; matteo.boattini@gmail.com; 3 Department of Public Health and Paediatrics, University of Torino, 10126 Turin, Italy; 4 Lisbon Academic Medical Centre, 1000-001 Lisbon, Portugal; 5 Microbiology and Virology Unit, Department of Pathology, Azienda Ospedaliera Universitaria Integrata di Verona, 37121 Verona, Italy; paolo.gaibani@univr.it; 6 Department of Diagnostic and Public Health, Microbiology Section, University of Verona, 37121 Verona, Italy; 7 Department of Experimental Medicine, University of Salento, 73100 Lecce, Italy; 8 Microbiology and Virology Unit, Vito Fazzi Hospital, 73100 Lecce, Italy

**Keywords:** carbapenem sparing, cefepime/enmetazobabtam, cefepime/taniborbactam, cefepime/zidebactam, metallo-β-lactamase, multi-drug-resistance, novel β-lactamase inhibitor, KPC, OXA-48-like, resistance mechanism

## Abstract

Cefepime combined with late-generation β-lactamase inhibitors—enmetazobactam, zidebactam, and taniborbactam—represents a promising strategy to treat multidrug-resistant Gram-negative infections. These combinations expand the therapeutic armamentarium beyond established β-lactam/β-lactamase inhibitor regimens, offering targeted activity against ESBL-, AmpC-, and carbapenemase-producing Enterobacterales, as well as multidrug-resistant *Pseudomonas aeruginosa*. In vitro studies highlight potent and broad activity, with mechanisms including β-lactamase inhibition and, in the case of zidebactam, dual β-lactam enhancement through PBP2 binding. Clinical evidence demonstrates efficacy in complicated urinary tract infections and suggests potential for treating extensively drug-resistant infections, including those unresponsive to conventional β-lactam/β-lactamase inhibitors. Emerging resistance mechanisms—such as PBP alterations, porin loss, efflux pump overexpression, and evolving KPC or NDM variants—underscore the need for ongoing surveillance and robust susceptibility testing. This review provides a comprehensive overview of the mechanisms of action, in vitro activity, pharmacokinetic/pharmacodynamic properties, clinical outcomes, and resistance patterns of these cefepime-based combinations. It also highlights future directions, including the establishment of clinical breakpoints, evaluation in severe infections, and exploration of combination strategies to counteract complex resistance. Overall, these agents exemplify a strategic evolution in β-lactam therapy, offering versatile options to reduce carbapenem reliance while maintaining high efficacy against multidrug-resistant Gram-negative pathogens.

## 1. Introduction

The worldwide rise in antimicrobial resistance among Gram-negative bacteria poses a serious challenge to contemporary medicine. β-lactam antibiotics continue to serve as the cornerstone of antibacterial therapy, accounting for approximately 65% of global antimicrobial consumption. Their widespread use is driven by broad-spectrum activity, a favorable safety profile, and well-established pharmacokinetic/pharmacodynamic (PK/PD) properties [1]. Nevertheless, the clinical utility of β-lactams is increasingly undermined by the spread of β-lactamases—enzymes that hydrolyze the β-lactam ring and render these agents ineffective.

Of particular concern is the global dissemination of extended-spectrum β-lactamases (ESBLs)—including TEM-, SHV-, and CTX-M-type variants—together with carbapenemases. The latter comprise Ambler class A serine carbapenemases (e.g., KPC), class B metallo-β-lactamases (MBLs; e.g., NDM, VIM, IMP), and class D oxacillinases (e.g., OXA-48-like and OXA-23-like). Collectively, these β-lactamases have markedly compromised the efficacy of β-lactam antibiotics and substantially narrowed therapeutic options for Gram-negative infections [2,3,4].

Carbapenems have therefore become the preferred treatment for severe infections caused by multidrug-resistant Gram-negative bacteria. However, their extensive use has created substantial selective pressure, accelerating the emergence of carbapenem-resistant Enterobacterales (CRE), carbapenem-resistant *Pseudomonas aeruginosa*, and carbapenem-resistant *Acinetobacter baumannii*. These pathogens are associated with high mortality and limited therapeutic options [5,6]. Consequently, international health organizations and antimicrobial stewardship programs have emphasized the urgent need for effective carbapenem-sparing strategies [7].

In this context, cefepime represents an attractive backbone for combination therapy. Cefepime has enhanced stability against chromosomal AmpC β-lactamases, high affinity for penicillin-binding proteins (PBPs), and favorable pharmacokinetic/pharmacodynamic (PK/PD) properties, including reliable tissue penetration [8]. However, cefepime remains susceptible to hydrolysis by ESBLs and carbapenemases, which limits its activity against contemporary multidrug-resistant pathogens.

The development of late-generation β-lactamase inhibitors (BLIs) has renewed interest in cefepime-based combinations. These agents restore cefepime activity, broaden its antibacterial spectrum, and offer carbapenem-sparing alternatives for both empiric and targeted therapy [9]. Each combination is designed to address distinct resistance mechanisms and clinical needs (Table 1). Cefepime/enmetazobactam was developed for the treatment of invasive infections, particularly complicated urinary tract infections (cUTIs) and bacteremia, caused by ESBL-producing Enterobacterales and has demonstrated superior efficacy compared with piperacillin–tazobactam in this setting [10,11]. Cefepime/taniborbactam combines cefepime with a bicyclic boronate inhibitor active against Ambler class A, B, C, and D β-lactamases, including some metallo-β-lactamases (MBLs) such as NDM and VIM [12]. Finally, cefepime/zidebactam employs a β-lactam enhancer strategy based on complementary PBP binding and the β-lactamase inhibitory activity of zidebactam (except against MBLs), enabling activity against MBL-producing microorganisms despite the absence of direct MBL inhibition. [12,13,14].

In this review, we summarize the mechanisms of action and in vitro activity of these combinations against multidrug-resistant Gram-negative pathogens. We highlight emerging resistance mechanisms and synthesize the available clinical data on their use and efficacy. Key PK/PD considerations for optimizing cefepime-based regimens in clinical practice are also discussed.

## 2. Literature Search and Study Selection

A comprehensive literature search was conducted to identify studies reporting on cefepime combined with late-generation β-lactamase inhibitors, including enmetazobactam, zidebactam, and taniborbactam. The databases searched included PubMed/MEDLINE, Embase, and Web of Science, covering publications up to January 2026. Search terms combined drug names with keywords related to β-lactamase inhibition, multidrug-resistant Gram-negative infections, in vitro activity, PK/PD, clinical trials, and resistance mechanisms. Studies were included if they reported original data on in vitro susceptibility, resistance, PK/PD analyses, in vivo efficacy, or clinical outcomes. Review articles and studies not providing primary data were excluded. While a formal PRISMA framework was not applied, study selection followed a structured approach, including screening of titles and abstracts, full-text review, and extraction of relevant data into standardized tables for qualitative synthesis.

## 3. Mechanisms of Action

**Figure 1:**
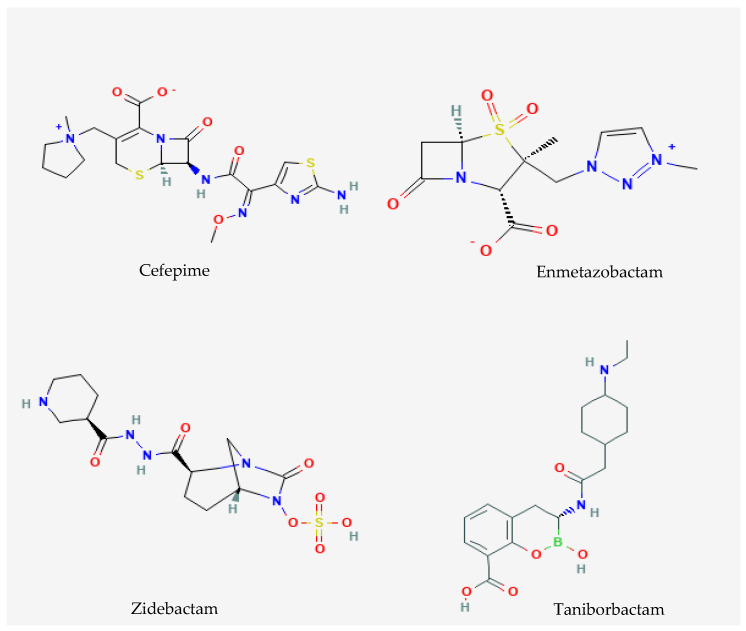
Chemical structures of cefepime and novel β-lactamase inhibitors analyzed in this review (https://pubchem.ncbi.nlm.nih.gov, accessed on 23 February 2026).

Cefepime is a fourth-generation cephalosporin with activity against Enterobacterales and *P. aeruginosa* [15]. Its zwitterionic structure enhances penetration across the outer membrane. Cefepime binds preferentially to PBP3, resulting in the inhibition of cell wall synthesis and rapid bactericidal activity (Figure 1). The drug is relatively stable against chromosomal AmpC β-lactamases. Its antibacterial efficacy is time-dependent and correlates with the proportion of the dosing interval during which free drug concentrations exceed the MIC (fT > MIC). Target attainment of approximately 60–70% fT > MIC has been associated with optimal outcomes, particularly in severe infections [16]. However, cefepime is readily hydrolyzed by ESBLs and carbapenemases, limiting its effectiveness as monotherapy against contemporary resistant pathogens. Consequently, combinations of cefepime with BLIs such as enmetazobactam, zidebactam, and taniborbactam have been developed to restore and expand its antibacterial activity. These novel agents substantially broaden the spectrum of cefepime, conferring activity against Enterobacterales producing ESBLs, AmpC enzymes, serine-carbapenemases, and, in selected combinations, MBLs. As such, the cefepime/BLI combination represents a promising carbapenem-sparing strategy for the treatment of multidrug-resistant Gram-negative infections (Table 1 and Table 2).

Enmetazobactam (formerly AAI101, Allecra Therapeutics) is a penicillanic acid sulfone β-lactamase inhibitor developed to enhance the inhibition of class A serine β-lactamases, particularly ESBLs prevalent among Enterobacterales. It exerts time-dependent, irreversible inhibition by forming a stable covalent acyl–enzyme complex that prevents β-lactam hydrolysis [10,12]. Structural optimization, including a strategically positioned methyl group conferring a zwitterionic charge, improves bacterial cell penetration, increases affinity for target enzymes, and prolongs residence time compared with earlier inhibitors (Figure 1). This modification enables additional hydrogen-bonding interactions within the CTX-M-15 active site and results in potent inhibition of CTX-M, TEM, SHV, and other class A enzymes (Table 2). Enmetazobactam shows limited activity against class C AmpC enzymes and lacks activity against class B MBLs and most class D carbapenemases (Table 2). However, cefepime is intrinsically stable to hydrolysis by many AmpC and OXA enzymes, making it an optimal partner for enmetazobactam [10,12]. The combination restores cefepime activity against ESBL-producing pathogens, supporting a carbapenem-sparing strategy [17]. As with most BLIs, enmetazobactam has no intrinsic antibacterial activity, and its clinical benefit derives from protection of the companion β-lactam.

Zidebactam (formerly WCK 5107; Wockhardt, Mumbai, India) is a diazabicyclooctane compound with a dual mechanism of action (Figure 1). It inhibits class A and class C serine β-lactamases through reversible covalent binding and, uniquely, binds with high affinity to PBP2, providing intrinsic antibacterial activity [13,14]. For this reason, zidebactam is considered a β-lactam enhancer rather than a conventional β-lactamase inhibitor. When combined with cefepime, which targets PBP3, complementary engagement of PBP2 and PBP3 results in rapid bactericidal activity, even at sub-MIC concentrations. This PBP-driven mechanism operates independently of β-lactamase expression and enables cefepime/zidebactam to remain active against organisms producing MBLs, despite the lack of direct class B enzyme inhibition [12,14]. Zidebactam also inhibits several class D enzymes in addition to class A and C β-lactamases (Table 2) [12,14]. Consequently, the combination offers a therapeutic option for infections caused by Gram-negative bacilli resistant to cefepime alone, including KPC- and MBL-producing carbapenem-resistant isolates and other multidrug-resistant pathogens [12].

Taniborbactam (formerly AAI101 VNRX-5133, Venatorx Pharmaceuticals, Malvern, PA, USA) is a bicyclic boronate β-lactamase inhibitor with an exceptionally broad inhibitory spectrum. Unlike avibactam, which selectively inhibits serine β-lactamases, taniborbactam incorporates an aromatic carboxylated moiety on the boronate ring that enables binding to MBLs (Figure 1). Steady-state kinetic analyses identified taniborbactam as a competitive inhibitor of VIM-2 and NDM-1, but not IMP-1, with inhibition constants (Ki) of 0.019, 0.081, and 30 μM, respectively [12,13]. The compound also displayed potent activity against class A, class C, and OXA-48–like class D enzymes, showing Ki values comparable to those of avibactam. Mechanistically, taniborbactam inhibits serine β-lactamases through slow dissociation, whereas it acts as a reversible competitive inhibitor with low Ki and rapid dissociation from MBLs, although it does not inhibit IMP-type MBLs [12,13]. When combined with cefepime, taniborbactam protects the cephalosporin from hydrolysis across a wide range of resistance determinants. This significantly expands antibacterial coverage and supports its potential role in the treatment of infections caused by carbapenemase-producing Gram-negative pathogens (Table 2) [18].

## 4. In Vitro Activity

### 4.1. Cefepime/Enmetazobactam

MIC determination for cefepime/enmetazobactam can be performed by broth microdilution in accordance with ISO 20776-1 using unsupplemented cation-adjusted Mueller–Hinton broth and a fixed enmetazobactam concentration of 8 mg/L [12]. In 2023, EUCAST introduced clinical breakpoints for cefepime/enmetazobactam (version 13.0), defining susceptibility for Enterobacterales as S ≤ 4 mg/L and resistance as R > 4 mg/L, with testing performed at the fixed inhibitor concentration of 8 mg/L. Following FDA approval in the same year, the FDA established clinical breakpoints for Enterobacterales of S ≤ 8 mg/L and R > 8 mg/L. To date, CLSI has not defined interpretive criteria for cefepime/enmetazobactam; therefore, EUCAST or FDA breakpoints are currently applied for in vitro interpretation. For *P. aeruginosa*, only FDA breakpoints are presently available (S ≤ 8 mg/L).

Large surveillance studies demonstrate the potent activity of cefepime/enmetazobactam against Enterobacterales, particularly ESBL producers (Table 3) [19,20,21,22,23,24,25,26,27,28]. In clinical isolates from the USA and Europe (2014–2015), MIC50/90 were 0.06/0.25 mg/L with 97.3% and 98.1% susceptibility by EUCAST and FDA criteria; ESBL-producing *Escherichia coli* and *Klebsiella pneumoniae* showed ≥98% susceptibility [19]. Comparable European data (2019–2021) confirmed full susceptibility among ESBL and AmpC isolates with MIC90 ≤ 1 mg/L [26]. An Indian multicentre survey (2023–2024) reported 100% susceptibility in ESBL/AmpC *E. coli* and *K. pneumoniae* with MIC90 ≤ 0.12 mg/L [26]. Belley et al., analyzing >7000 isolates (2016–2018), documented 98.8% susceptibility by FDA and near-universal activity against third-generation cephalosporin-resistant Enterobacterales [21].

Activity against carbapenemase-producing Enterobacterales (CPE) is enzyme-dependent (Table 3). Tselepis et al. showed poor activity against KPC (MIC90 64 mg/L) but better results for OXA-48-like isolates [20]. Although cefepime/enmetazobactam demonstrates limited residual activity against KPC-producing isolates, recent studies have reported potent antimicrobial efficacy against KPC variants associated with resistance to ceftazidime/avibactam [29]. Moreover, in OXA-48–producing strains, in vitro data indicate that enmetazobactam significantly enhances the bactericidal activity of cefepime [30]. A Spanish multicentre study of 400 CPE reported 67.8% EUCAST susceptibility overall, rising to 74% for OXA-48-like producers, while KPC and MBL isolates showed only 40.9% and 3.6% of susceptibility, respectively [10]. French surveillance (2021–2023) confirmed high activity for OXA-48-like isolates (MIC90 2 mg/L; 98.8% susceptible) but minimal effect against NDM (0.9%) and variable results for VIM (60.6%) and KPC (63.3%) producers [24]. Dutkiewicz et al. further documented 94.1% susceptibility for OXA-48, 30.2% for VIM, 3% for NDM, and complete resistance in NDM+OXA-48 co-producers [28].

In carbapenem-non-susceptible but non-CPE, activity is intermediate. Taiwanese data showed 84.6% susceptibility overall with elevated MICs in *K. pneumoniae* [23], while a French evaluation of ESBL/AmpC non-carbapenemase-producing isolates reported 68.9% susceptibility [27], indicating partial restoration of cefepime activity beyond classical ESBL phenotypes.

Evidence for non-fermenting Gram-negative species is limited (Table 3). Initial studies in *P. aeruginosa* showed MIC90 16 mg/L with ~83% susceptibility [19]. Among carbapenem-resistant Taiwanese isolates, susceptibility decreased to 58% with MIC90 32 mg/L [22], whereas unselected Indian isolates displayed MIC90 8 mg/L and 100% susceptibility by FDA criteria [26], reflecting geographic differences in resistance mechanisms.

Overall, cefepime/enmetazobactam provides consistent coverage of ESBL- and AmpC-producing Enterobacterales across regions [19,21,25,26]. The main limitation is reduced efficacy against non-OXA-48 carbapenemases, particularly MBLs [10,24,28]. Activity against *P. aeruginosa* remains variable and epidemiology-dependent [19,22,26].

### 4.2. Cefepime/Zidebactam

The in vitro activity of cefepime/zidebactam (FEP/ZID) against Gram-negative pathogens is summarized in Table 4, which compiles data from global and regional surveillance studies and focused collections of clinical Gram-negative isolates [10,22,28,31,32,33,34,35,36,37,38,39]. MICs of cefepime/zidebactam can be determined by broth microdilution in accordance with ISO 20776-1 using unsupplemented cation-adjusted Mueller–Hinton broth, with cefepime and zidebactam tested at a fixed 1:1 concentration ratio [12]. At present, no official clinical breakpoints are available for the cefepime/zidebactam combination. Consequently, susceptibility results across studies have been interpreted using cefepime breakpoints established by EUCAST or CLSI, or alternatively by applying provisional breakpoints derived from PK/PD analyses.

Overall, FEP/ZID demonstrated potent in vitro activity against Enterobacterales. Large-scale worldwide surveillance studies reported very low MIC distributions, with MIC50 values typically ≤0.12–0.25 mg/L and MIC90 values ≤1–2 mg/L for unselected Enterobacterales isolates, resulting in susceptibility rates exceeding 99% when EUCAST or CLSI cefepime breakpoints were applied [14,31,35]. High activity was also maintained against extended-spectrum β-lactamase (ESBL)-producing isolates, with susceptibility rates close to 100% across multiple studies [34,35]. Importantly, cefepime/zidebactam retained substantial activity against CRE isolates. In global collections, MIC90 values for CRE generally ranged from 2 to 4 mg/L, with susceptibility rates above 90% according to EUCAST criteria, though lower rates were observed when CLSI breakpoints were applied [14,35,39]. Activity was particularly pronounced against KPC- and OXA-48-like–producing isolates, for which MIC90 values remained consistently low (≤1–2 mg/L) and susceptibility rates frequently reached 100% [10,34,37,39]. Notably, in vitro data demonstrated that cefepime/zidebactam retained high antimicrobial activity against *Klebsiella pneumoniae harboring bla_KPC_* variants associated with resistance to ceftazidime/avibactam [12,40]. By contrast, activity against MBL-producing Enterobacterales, including NDM- and VIM-producing strains, was more variable. Although MIC50 values remained relatively low in several studies, MIC90 values increased substantially (up to 16–32 mg/L), resulting in reduced susceptibility rates when standard cefepime breakpoints were used [33,34,38]. The application of provisional PK/PD breakpoints, however, suggested preserved activity in a significant proportion of these isolates [33,36,37].

Against *P. aeruginosa*, cefepime/zidebactam exhibited consistently strong activity. In worldwide surveillance studies, MIC90 values were generally 4–8 mg/L, with susceptibility rates around 97–99% using EUCAST or CLSI criteria [14,31,35]. Notably, FEP/ZID maintained activity against carbapenem-resistant and multidrug-resistant *P. aeruginosa*, including isolates non-susceptible to meropenem, piperacillin/tazobactam, ceftazidime/avibactam, or ceftolozane/tazobactam, although modest increases in MICs and lower susceptibility rates were observed in these subsets [33,34,35]. Reduced activity was primarily associated with MBL production or markedly increased efflux expression [33,34]. In contrast, activity against *A. baumannii* was limited. Across multiple studies, MIC90 values were high (32–64 mg/L), and susceptibility rates were low when interpreted using CLSI or PK/PD breakpoints [14,35]. Some residual activity was observed against OXA-type carbapenemase-producing isolates, whereas FEP/ZID showed minimal to no activity against MBL-producing *A. baumannii* [34,35]. Finally, cefepime/zidebactam displayed variable activity against *Stenotrophomonas maltophilia*, with MICs generally higher than those observed for Enterobacterales and *P. aeruginosa*, and no established breakpoints to support categorical interpretation [33,34,35]. Collectively, these data indicate that cefepime/zidebactam has excellent in vitro activity against Enterobacterales, including many carbapenem-resistant phenotypes, and robust activity against *P. aeruginosa*, while its role against *A. baumannii* appears limited, underscoring the need for dedicated breakpoints and further clinical evaluation.

### 4.3. Cefepime/Taniborbactam

MICs of cefepime/taniborbactam can be determined by broth microdilution in accordance with ISO 20776-1 using unsupplemented cation-adjusted Mueller–Hinton broth, with taniborbactam fixed at 4 mg/L [12]. Currently, no official clinical breakpoints are available for cefepime/taniborbactam. Consequently, studies have generally interpreted susceptibility results according to cefepime breakpoints from EUCAST and CLSI or using provisional PK/PD-based breakpoints [10,28,37,38,41,42,43,44,45,46,47,48].

Across multiple studies, cefepime/taniborbactam consistently showed potent in vitro activity against Enterobacterales, particularly those producing class A and D carbapenemases (Table 5). In the Indian multicenter study by Bakthavatchalam et al. [37], susceptibility rates among *E. coli* and *K. pneumoniae* isolates varied markedly depending on carbapenemase type. While OXA-48-like producers remained highly susceptible (≥95%), activity was reduced against NDM- and NDM+OXA-48-like co-producers, with susceptibility ranging from 5 to 20% (MIC_50_ 32 mg/L). Similarly, Liu et al. [38] in China reported high MICs for NDM-producing Enterobacterales (MIC_50_ 2 mg/L; MIC_90_ 32 mg/L), highlighting limited efficacy against MBL enzymes.

In contrast, activity against KPC and OXA-48 producers was consistently high across geographies. In the global GEARS surveillance (2018–2020) [44], cefepime/taniborbactam inhibited 99.7% of Enterobacterales overall, with excellent activity against KPC (100%) and OXA-48 (98.8%) strains, and good susceptibility among carbapenem-resistant (94.5%) or carbapenemase-producing (94.6%) isolates. Concordant findings were observed in European studies by Hernández-García et al. [45] (Spain, 2020; 97.6% susceptible overall) and by Dutkiewicz et al. [28] (France, 2024), where susceptibility reached 100% for KPC, 98.3% for VIM, and 97.6% for OXA-48 producers, but declined to 76% and 26% for NDM-producing and NDM+OXA-48-co-producing Enterobacterales, respectively.

Similar patterns were reported by Meletiadis et al. [42] in Greece, where cefepime/taniborbactam achieved MIC50/90 values of 2/16 mg/L and susceptibility of 78% among mβL-producing *K. pneumoniae*. Studies involving ESBL- and AmpC-producing isolates demonstrated near-universal susceptibility, with MIC90 ≤1 mg/L and 100% susceptible across multiple collections from China and Europe [41,43,44].

The activity against *P. aeruginosa* was moderately effective but variable across studies. Karlowsky et al. [44] reported high activity globally (97.4% overall; 92.4% among carbapenem-resistant isolates), although MBL-carrying strains showed reduced susceptibility (66.5%). Similarly, Hernández-García et al. [45] observed 67.5% overall susceptibility in Spanish isolates, dropping to 61% in VIM producers. Data from Jacobs et al. [46] in the USA indicated 82.7% susceptibility overall, but only 35% against KPC-producing *P. aeruginosa*. Asian data showed comparable trends with 70.6% activity against carbapenem-non-susceptible isolates in Taiwan [48], whereas Meletiadis et al. [42] observed only 46% susceptibility among mβL-positive strains in Greece. Limited information exists for *Acinetobacter* species. In the Taiwanese collection [48], *A. baumannii* isolates were uniformly resistant (MIC50/90 > 32 mg/L, 5.2% susceptibility), consistent with the expected lack of activity against this genus. Large surveillance studies reinforce these findings. Karlowsky et al. [44] and Santerre Henriksen et al. [47] confirmed global uniform efficacy against Enterobacterales (>98%) and carbapenem-resistant subsets (≈93%), emphasizing taniborbactam’s broad activity across most β-lactamase classes.

Overall, the data indicate that cefepime/taniborbactam exhibits potent in vitro activity against Enterobacterales producing KPC, OXA-48-like, ESBL, or AmpC enzymes, and notable though variable efficacy against *P. aeruginosa*. However, activity against MβL- and NDM-producing isolates remains geographically variable. This finding is explained by the geographical differences in the distribution of bacterial clones harboring resistance mechanisms, including the expression of specific β-lactamase variants [12]. The use of cefepime clinical or PK/PD-derived breakpoints across studies underscores the current need for harmonized interpretive criteria to better define resistance thresholds and guide future clinical application.

## 5. Pharmacokinetics/Pharmacodynamics

### 5.1. Cefepime/Enmetazobactam

The antibacterial efficacy of cefepime/enmetazobactam depends on the attainment of joint PK/PD targets that consider both the β-lactam and the β-lactamase inhibitor components. Preclinical models demonstrated that maintenance of enmetazobactam concentrations around 2 mg/L is required to restore cefepime activity against ESBL-producing Enterobacterales, preventing resistance amplification and enabling sustained bacterial killing [49]. In hollow-fiber experiments simulating human exposures with cefepime 2 g/enmetazobactam 0.5 g every 8 h as a 2 h infusion, significant bacterial reductions were achieved for isolates expressing ESBLs alone, whereas co-producing carbapenemases showed limited response when MIC values were ≥4 mg/L [50]. Murine thigh and lung infection models further clarified the PK/PD determinants. For isolates with MICs up to 2 mg/L, bactericidal activity was associated with cefepime free concentrations above the MIC for 40–60% of the dosing interval and with enmetazobactam free concentrations >2 mg/L for approximately 44% of the interval [49]. In pneumonia models, a ≥2-log_10_ reduction in lung burden was observed when both cefepime and enmetazobactam achieved at least 20% fT > MIC and 20% fT > 2 mg/L in plasma and epithelial lining fluid (ELF) [51]. Importantly, cefepime/enmetazobactam showed superior in vivo activity compared with meropenem against OXA-48–producing *K. pneumoniae* despite low meropenem MICs [52]. ELF penetration in healthy volunteers averaged 60% for cefepime and 53% for enmetazobactam, supporting their use in respiratory infections [53].

Population PK analyses from phase 1–3 studies revealed similar disposition of the two agents, with volumes of distribution around 20–21 L, limited protein binding, and predominant renal elimination as unchanged drug [54,55]. Monte-Carlo simulations based on these data predicted >95% probability of target attainment (PTA) at day 7 for the approved regimen against Enterobacterales with MICs up to 8 mg/L [25]. To reach more stringent pulmonary targets (60% fT > MIC for cefepime and 45% fT > 2 mg/L for enmetazobactam), extension of the infusion to 4 h is recommended in patients with pneumonia or augmented renal clearance [55]. The clinical PK of cefepime is characterized by wide inter-patient variability, particularly in critically ill subjects, those with augmented renal clearance, or receiving renal replacement therapies [8,56,57]. Neurotoxicity risk correlates with excessive exposure, with trough concentrations above 35–45 mg/L identified as a safety threshold, highlighting the potential role of therapeutic drug monitoring in complex patients [58,59]. Enmetazobactam displays comparable renal elimination with 90% urinary recovery and requires dose reduction in renal impairment, while no adjustment is needed for hepatic dysfunction or advanced age [54].

Overall, available PK/PD evidence supports the licensed dosing of cefepime/enmetazobactam 2 g/0.5 g every 8 h infused over 2 h, with prolongation to 4 h in selected scenarios. The combination achieves robust exposures in plasma and ELF, retains activity against ESBL and some OXA-48 producers, and its pharmacodynamic profile appears unaffected by pulmonary surfactant [60]. These characteristics provide a strong rationale for its use in severe infections due to multidrug-resistant Enterobacterales.

### 5.2. Cefepime/Zidebactam

Zidebactam displays a limited volume of distribution (approximately 15–20 L), low plasma protein binding (<15%), and a short elimination half-life, features consistent with other BLIs [61]. After repeated administration of cefepime/zidebactam 2/1 g every 8 h, intrapulmonary penetration was documented with ELF to plasma ratios around 38% and alveolar macrophage penetration close to 10% [62]. Renal function represents the main determinant of systemic exposure. A dedicated study including subjects with varying degrees of renal impairment demonstrated a progressive reduction in renal clearance and a parallel increase in AUC and half-life for both components, supporting the need for dose adjustment in moderate to severe dysfunction and in patients receiving intermittent hemodialysis [63]. These findings are consistent with the predominantly renal elimination of both agents and the absence of relevant non-renal metabolic pathways.

The pharmacodynamic behavior of the combination differs from that of cefepime alone. In neutropenic murine pneumonia models caused by MBL–producing Enterobacterales, a strong correlation between cefepime %fT > MIC in the presence of zidebactam and bacterial killing was observed (R^2^ ≈ 0.82). Remarkably, net bacterial stasis was achieved at cefepime exposures as low as 18% fT > MIC, while a 1-log_10_ reduction required approximately 31% fT > MIC, values considerably lower than classical cephalosporin targets [64]. Similar enhancement was confirmed against *A. baumannii*, where the addition of zidebactam reduced the cefepime exposure needed for a 1-log_10_ kill from nearly 40% to about 15% fT > MIC [65].

Human-simulated exposure studies further supported these observations. In murine lung and thigh infection models involving carbapenem-resistant *A. baumannii* and multidrug-resistant *P. aeruginosa*, cefepime/zidebactam consistently produced significant reductions in bacterial burden, whereas either agent alone was largely ineffective [63,66]. The activity was maintained across isolates with elevated cefepime MICs, indicating that the enhancer effect of zidebactam can overcome traditional resistance mechanisms.

Translational models employing plasma and ELF humanized profiles showed that clinically achievable regimens resulted in ≥1-log_10_ reductions against KPC- and OXA-48–producing *K. pneumoniae* in pneumonia models, with comparable efficacy between plasma and ELF exposures [67]. Collectively, these data suggest that the PK/PD driver for cefepime/zidebactam relies on the synergistic interaction between PBP2 engagement by zidebactam and time-dependent cefepime activity, allowing effective bacterial killing at lower cefepime exposures than historically required.

Overall, the available evidence indicates that cefepime/zidebactam possesses favorable pharmacokinetic properties, predictable behavior in renal impairment, and a distinctive pharmacodynamic profile capable of restoring cefepime efficacy against challenging Gram-negative pathogens, including MBL-producing Enterobacterales, carbapenem-resistant *A. baumannii*, and multi-drug-resistant *P. aeruginosa*.

### 5.3. Cefepime/Taniborbactam

First-in-human studies demonstrated dose-proportional pharmacokinetics after 2 h intravenous infusions, with low interindividual variability and linear exposure across single and multiple dosing regimens [68]. Renal excretion represents the main elimination pathway; clearance decreases by approximately 15%, 63%, and 81% in mild, moderate, and severe renal impairment, respectively, and the compound is efficiently removed by hemodialysis, supporting the need for renal-adjusted dosing [69].

Pulmonary disposition has been investigated following administration of cefepime/taniborbactam 2/0.5 g over 2 h. Mean taniborbactam exposure in ELF corresponds to about 18% of plasma concentrations, whereas alveolar macrophage penetration approaches 95%, indicating extensive intracellular distribution [70]. Similar ELF/plasma ratios ranging from 15% to 25% were confirmed in an independent study, supporting predictable lung exposure [71]. When the combination was infused over 4 h, unbound ELF concentrations of taniborbactam remained within pharmacodynamically relevant ranges throughout the dosing interval [71].

The pharmacodynamic drivers of activity appear to involve both time- and exposure-dependent parameters. In vitro dynamic models identified taniborbactam AUC and %fT > MIC as key predictors of cefepime potentiation, with a taniborbactam AUC of approximately 4–11 mg·h/L required for a 1-log_10_ reduction in bacterial burden [72]. Murine thigh and pneumonia models further refined these targets. Against serine-β-lactamase–producing Enterobacterales, median taniborbactam fAUC_0–24_/MIC values associated with 1-log kill ranged around 2–4, while lower targets were observed for *P. aeruginosa* [73,74]. In resistant urinary tract and lung infection models, human-simulated exposures of cefepime/taniborbactam achieved ≥1–2 log_10_ reductions against isolates harboring ESBL, AmpC, KPC, OXA-48, and MBLs, whereas cefepime alone was largely ineffective [74,75].

Hollow-fiber infection experiments simulating the clinical regimen of 2/0.5 g every 8 h for 7 days demonstrated rapid bactericidal activity against diverse cefepime-resistant Enterobacterales and *P. aeruginosa*. Importantly, no emergence of resistant subpopulations was detected, suggesting a high barrier to resistance when both agents are co-administered [76]. Additional murine PK/PD analyses indicated that taniborbactam %fT above a threshold concentration best described efficacy at standard cefepime exposures, whereas fAUC became more relevant at higher cefepime levels, reflecting a dual mechanism of potentiation [77].

Collectively, these data indicate that cefepime/taniborbactam achieves adequate systemic and pulmonary exposures with predictable behavior in renal dysfunction. The combination restores cefepime activity against a broad spectrum of β-lactamase–mediated resistance mechanisms, with pharmacodynamic targets attainable using the proposed clinical regimen of 2/0.5 g every 8 h infused over 2–4 h. Although human PK/PD–outcome correlations remain limited, preclinical evidence consistently supports its potential role in the treatment of severe infections due to multidrug-resistant Gram-negative pathogens.

## 6. Clinical Evidences

### 6.1. Cefepime/Enmetazobactam

The in vivo efficacy of cefepime/enmetazobactam has been primarily established through a structured clinical development program. Early-phase studies supported dose selection and PK/PD optimization, demonstrating predictable systemic exposure and renal elimination of both agents, as well as favorable penetration into ELF, supporting extrapolation to pulmonary infections [51,53]. 

Clinical efficacy data are largely derived from the ALLIUM trial, a Phase III, double-blind, multicenter, non-inferiority study comparing cefepime/enmetazobactam with piperacillin/tazobactam in adult patients with cUTIs, including acute pyelonephritis [78].

A total of 1041 patients were randomized, and 1034 received at least one dose of study drug (modified intention-to-treat population). Patients received cefepime/enmetazobactam 2 g/0.5 g every 8 h administered as a 2 h intravenous infusion, with dose adjustment in moderate renal impairment. The comparator arm received piperacillin/tazobactam 4 g/0.5 g every 8 h. The primary endpoint was overall treatment success at the test-of-cure (TOC) visit (days 14–21), defined as a composite of clinical cure and microbiological eradication in the microbiological modified intention-to-treat (mMITT) population. Cefepime/enmetazobactam demonstrated both non-inferiority and statistical superiority over piperacillin/tazobactam. Overall treatment success at TOC was achieved in 79.1% of patients receiving cefepime/enmetazobactam compared with 58.9% in the comparator arm, yielding an absolute difference of more than 20%, which was both statistically significant and clinically meaningful. Superiority was maintained at the late follow-up visit (days 21–28), supporting sustained microbiological response beyond completion of therapy [78]. Importantly, clinical cure rates alone were high and comparable between groups, whereas superiority was primarily driven by significantly higher microbiological eradication rates in the cefepime/enmetazobactam arm. Microbiological eradication was achieved in over 80% of treated patients, compared with approximately two-thirds of those receiving piperacillin/tazobactam [78]. This effect was consistent across the most frequent Enterobacterales pathogens, including *E. coli*, *K. pneumoniae*, *Proteus mirabilis*, and *Enterobacter cloacae*. Efficacy advantages were particularly evident in infections caused by extended-spectrum β-lactamase (ESBL)-producing Enterobacterales. In this clinically relevant subgroup, cefepime/enmetazobactam achieved substantially higher overall success rates than piperacillin/tazobactam, underscoring the contribution of enmetazobactam to restoring cefepime activity against resistant phenotypes [54,78]. In parallel, microbiological recurrence rates were markedly lower in patients treated with cefepime/enmetazobactam, suggesting more complete pathogen clearance. Subgroup analyses showed consistent efficacy across age groups, sex, baseline renal function, infection type, comorbidity burden, and presence of baseline bacteremia. Although only a limited number of patients with *P. aeruginosa* infection were included, overall outcomes in this subgroup were similar between treatment arms [78].

Emergence of reduced susceptibility during therapy was infrequent. A small number of post-baseline isolates exhibited increases in cefepime/enmetazobactam minimum inhibitory concentrations, with only one isolate showing a categorical shift; no clear resistance mechanisms were identified [79]. These findings suggest a low short-term risk of resistance development under clinical conditions.

While no dedicated Phase III trials have evaluated cefepime/enmetazobactam in pneumonia or bacteremia, regulatory approval in Europe and the UK for these indications was supported by PK/PD extrapolation and robust preclinical in vivo data. Murine pneumonia models demonstrated effective bacterial killing against ESBL-producing Enterobacterales, consistent with drug exposure achieved in the lung and ELF [51,55]. Collectively, available in vivo evidence supports the clinical efficacy of cefepime/enmetazobactam in cUTIs and provides a mechanistic and translational rationale for its use in other serious Gram-negative infections.

### 6.2. Cefepime/Zidebactam

The in vivo performance of cefepime/zidebactam has been explored through early clinical trials and an increasing body of real-world experiences, mainly involving infections caused by extensively drug-resistant (XDR) *P. aeruginosa* and other difficult-to-treat Gram-negative pathogens. Phase I investigations evaluated safety and pharmacokinetics in healthy volunteers and in subjects with renal impairment, demonstrating predictable exposure and the need for dose adjustment according to renal function [61]. A phase III randomized trial in cUTIs and acute pyelonephritis has been completed, comparing cefepime/zidebactam with meropenem; however, outcome data have not yet been released (Table 1) [80]. Despite the limited availability of controlled efficacy studies, multiple compassionate-use reports provide relevant clinical insights. Successful use of cefepime/zidebactam as a bridge to liver transplantation was described in a patient with cholangitis due to IMP-positive *P. aeruginosa* and NDM-5/OXA-232–producing *K. pneumoniae* that became resistant to cefiderocol during therapy. Both isolates remained susceptible to cefepime/zidebactam, and a 14-day course combined with source control allowed infection resolution and subsequent transplantation [81]. Similar encouraging outcomes have been reported in deep-seated infections. In a series of five patients with osteoarticular or endovascular XDR *P. aeruginosa* infections, treatment with cefepime/zidebactam for 2–6 weeks led to marked clinical and radiological improvement without drug-related adverse events, confirming the translational value of in vitro susceptibility [82].

Individual case reports further support the potential of the combination in highly challenging scenarios. A pediatric patient with refractory empyema caused by NDM-producing XDR *P. aeruginosa*, unresponsive to colistin and aztreonam plus ceftazidime/avibactam, achieved microbiological cure after 21 days of cefepime/zidebactam administered under compassionate access [83]. Another report described a renal-transplant recipient with sino-pulmonary infection and skull-base osteomyelitis due to XDR *P. aeruginosa*. Prolonged therapy guided by therapeutic drug monitoring resulted in near-complete radiological resolution, highlighting the importance of individualized dosing in sites with limited drug penetration [84]. Cefepime/zidebactam has also been used successfully in intra-abdominal sepsis caused by NDM-expressing *P. aeruginosa* resistant to all available β-lactam/β-lactamase inhibitor combinations. After failure of polymyxin-based therapy, administration of cefepime/zidebactam led to rapid clinical stabilization and hospital discharge, underscoring its role as salvage treatment for MBL-producers [85]. Likewise, disseminated infection in a patient with acute T-cell leukemia was controlled using the combination as last-line therapy, with sustained clinical response despite profound immunosuppression [86]. A recent multicenter Indian case series including deep-seated infections confirmed consistent effectiveness of cefepime/zidebactam against XDR *P. aeruginosa*, with favorable tolerability and absence of emergent resistance during therapy [87].

Collectively, available in vivo evidence, although largely observational, indicates that cefepime/zidebactam can achieve meaningful clinical responses in infections caused by organisms resistant to most current options, including MBL-producing *P. aeruginosa*. The dual mechanism of PBP2 engagement and β-lactamase inhibition appears to translate into therapeutic benefit across diverse anatomical sites. Ongoing phase III studies will be crucial to better define its comparative efficacy and optimal positioning, but present data already suggest a valuable role for cefepime/zidebactam in the management of severe infections due to XDR Gram-negative pathogens.

### 6.3. Cefepime/Taniborbactam

The clinical efficacy of cefepime/taniborbactam has been established in the Phase III CERTAIN-1 trial, a randomized, double-blind, multicenter study comparing cefepime/taniborbactam with meropenem for the treatment of cUTIs, including acute pyelonephritis (Table 1) [88,89]. In this global study, hospitalized adults were randomized in a 2:1 ratio to receive cefepime/taniborbactam (2.5 g every 8 h administered as a 2 h intravenous infusion) or meropenem (1 g every 8 h) for 7 days, with treatment extended up to 14 days in patients with concomitant bacteremia. Dose adjustments were performed according to renal function. The primary endpoint was composite success at the TOC visit, defined as the combination of clinical cure and microbiological eradication in the mITT population [88]. Cefepime/taniborbactam met the predefined non-inferiority margin versus meropenem and subsequently demonstrated statistical superiority. Composite success at TOC was achieved in 70.6% of patients receiving cefepime/taniborbactam compared with 58.0% in the meropenem group, corresponding to an absolute difference exceeding 12 percentage points [88]. The superiority signal was maintained at the late follow-up visit, supporting the durability of both clinical and microbiological responses.

As observed in other contemporary cUTI trials, clinical cure rates alone were high and generally similar between treatment arms, whereas differences in composite outcomes were primarily driven by higher microbiological eradication rates in the cefepime/taniborbactam group. Importantly, efficacy was consistent across key predefined subgroups, including stratification by infection type (cUTI vs. acute pyelonephritis), baseline renal function, age, sex, and presence of bacteremia [88].

A detailed microbiological subgroup analysis further strengthened the robustness of the findings [89]. High composite success rates were observed in patients infected with cefepime-resistant isolates, multidrug-resistant (MDR) pathogens, and carbapenem-resistant Enterobacterales (CRE). Favorable outcomes were documented across resistance genotypes, including isolates harboring ESBLs, AmpC enzymes, and carbapenemases. Notably, efficacy was maintained in infections caused by carbapenemase-producing organisms, including OXA-48-like, KPC, and NDM producers, providing important in vivo confirmation of taniborbactam’s broad β-lactamase inhibition profile [89]. Clinical efficacy against *P. aeruginosa* was also demonstrated. Although modest differences were observed in composite endpoints, clinical success rates were comparable to those achieved with meropenem, supporting activity in this challenging pathogen [89].

Cefepime/taniborbactam was generally well tolerated in clinical studies. The overall incidence of adverse events was similar to that observed with meropenem, with headache and gastrointestinal symptoms reported most frequently [18,88]. Dedicated pharmacokinetic studies in subjects with varying degrees of renal impairment confirmed predictable and parallel reductions in cefepime and taniborbactam clearance, supporting dose adjustment strategies without new safety concerns [69].

Although further Phase III studies are planned to expand the evidence base in severe infections such as ventilator-associated pneumonia, clinical development in this indication is currently on hold [90]. Overall, available in vivo data support cefepime/taniborbactam as an effective therapeutic option for cUTIs caused by highly resistant Gram-negative pathogens.

## 7. Resistance Mechanisms

### 7.1. Cefepime/Enmetazobactam

Resistance to cefepime/enmetazobactam primarily arises from the production of β-lactamases capable of hydrolyzing cefepime and not inhibited by enmetazobactam, particularly carbapenemases (Table 1). While enmetazobactam shows potent inhibition of ESBLs, it lacks activity against MBLs and most KPC variants [10,25]. OXA-48 producers without co-expressed ESBLs or plasmid-mediated AmpC may remain susceptible; however, the frequent accumulation of additional resistance mechanisms often compromises activity (Table 1) [24,25].

Reduced outer membrane permeability is a key contributor to resistance. Alterations or loss of OmpK35 and OmpK36 markedly decrease cefepime penetration, particularly in isolates already producing β-lactamases [10,91]. In carbapenemase-producing *K. pneumoniae*, amino acid substitutions or truncations in these porins have been strongly associated with elevated cefepime/enmetazobactam MICs, especially among OXA-48 and KPC producers [10,92,93].

Efflux-mediated resistance also plays a role. Overexpression of the AcrAB–TolC efflux system, frequently driven by mutations in regulatory genes such as ramR, has been associated with reduced susceptibility in ESBL- and OXA-48-co-producing isolates [10]. In addition, target modifications affecting cefepime binding have been described. Altered PBP3 expression or point mutations can contribute to resistance, and isolates in which SHV-1 hyperproduction was the sole detected mechanism have been reported [10]. While OXA-1 alone may increase cefepime MICs without exceeding resistance breakpoints, co-production with CTX-M-15 consistently results in resistance [10]. Overall, resistance to cefepime/enmetazobactam is typically multifactorial, reflecting the combined effects of β-lactamase production, porin loss, efflux pump overexpression, and target modification [10,93].

### 7.2. Cefepime/Zidebactam

Resistance to cefepime/zidebactam is largely driven by modifications of the primary zidebactam target, PBP2, reflecting the compound’s unique β-lactam enhancer mechanism (Table 1). In *E. coli*, specific substitutions in PBP2—most notably V522I—appear to be required for clinically relevant MIC increases, whereas insertions in PBP3 alone do not confer resistance to the combination [12,94].

Although zidebactam retains activity against most serine β-lactamases, collateral resistance has been described in *K. pneumoniae* carrying certain KPC variants related to the different types of modifications [95]. Although indels (i.e., insertions or deletions) have not been related to a lower activity of cefepime/zidebactam, single SNP mutations in the 270-loop region of KPC enzymes have been associated with reduced susceptibility, though these events appear less frequent than PBP-mediated resistance [95]. In MBL-producing Enterobacterales, resistance is more commonly associated with porin loss or structural alterations (e.g., OmpK35 and OmpC) rather than with impaired β-lactamase inhibition [12,96].

In *P. aeruginosa*, resistance development is typically multifactorial. Experimental evolution and in vitro studies have demonstrated that resistance requires the accumulation of mutations affecting PBP2 and PBP3, together with overexpression of the MexAB–OprM efflux system and alterations in its regulatory pathways [12,93,94,95,97,98]. Missense mutations within the transpeptidase domain of PBP2, particularly residues involved in direct zidebactam binding, significantly reduce enhancer activity and lead to increased MICs [97,99].

Importantly, the first case of treatment-emergent resistance to cefepime/zidebactam in vivo has recently been reported in a patient with *P. aeruginosa* pneumonia [100]. During therapy, cefepime/zidebactam MICs increased fourfold and were associated with clinical failure. Whole-genome sequencing identified newly acquired mutations in the MexAB–OprM efflux operon, which also conferred cross-resistance to ceftazidime/avibactam and imipenem/relebactam, underscoring the clinical relevance of efflux-driven resistance in *P. aeruginosa* [100].

### 7.3. Cefepime/Taniborbactam

Resistance to cefepime/taniborbactam is uncommon but mechanistically diverse, reflecting the broad inhibitory spectrum of taniborbactam against Ambler class A, B, C, and D β-lactamases (Table 1). One frequently described mechanism involves alterations in PBP3, particularly YRIN or YRIK insertions at positions P333_Y334, sometimes accompanied by substitutions such as E353K or I532L [92,97]. These changes reduce cefepime affinity but are generally insufficient alone to confer high-level resistance.

Porin alterations represent another important determinant. Loss or modification of OmpK35, OmpK36, OmpA, or regulatory elements such as OmpR has been associated with elevated MICs, particularly when combined with carbapenemase production [10,44,92]. Efflux pump upregulation mediated by mutations in regulators of the AcrAB system has also been reported in isolates with cefepime/taniborbactam MICs > 16 mg/L [10].

A critical emerging mechanism involves specific NDM variants. While taniborbactam retains potent activity against most NDM- and VIM-like enzymes, variants such as NDM-9, NDM-30, and the recently described NDM-60 exhibit reduced susceptibility [101,102,103,104]. Structural and biochemical studies have demonstrated that amino acid substitutions—most notably the replacement of Glu149 with Lys in NDM-9—significantly reduce taniborbactam binding affinity, providing a molecular basis for resistance [103]. Importantly, taniborbactam shows no activity against IMP-type MBLs, representing a consistent gap in its spectrum and highlighting a key limitation for MBL-producing Enterobacterales and *P. aeruginosa* [12]. The increasing dissemination of these variants raises concern regarding the long-term durability of taniborbactam against evolving MBLs.

In *P. aeruginosa*, elevated cefepime/taniborbactam MICs are usually associated with the accumulation of multiple chromosomal mechanisms, including IMP production, PBP3 mutations, efflux pump overexpression, and AmpC (PDC) derepression [44,45]. Collectively, available data indicate that high-level resistance to cefepime/taniborbactam generally requires the convergence of several resistance determinants, with production of specific NDM variants and IMP enzymes representing the most impactful single mechanisms identified to date.

## 8. Conclusions

Cefepime/enmetazobactam, cefepime/zidebactam, and cefepime/taniborbactam represent a significant advance in carbapenem-sparing strategies for multidrug-resistant Gram-negative infections. By expanding therapeutic options beyond established β-lactam/β-lactamase inhibitor combinations such as ceftazidime/avibactam, meropenem/vaborbactam, and imipenem/relebactam, these agents provide targeted activity against ESBL-, AmpC-, and CPE and multidrug-resistant *P. aeruginosa*, while potentially reducing selective pressure on carbapenems [105].

In vitro and in vivo data suggest a differentiated positioning of these combinations. Cefepime/enmetazobactam demonstrates consistent activity against ESBL- and AmpC-producing Enterobacterales, with more limited efficacy against non-OXA-48 carbapenemases and variable activity against *P. aeruginosa* [10,19,20,21,22,23,24,25,26,27,28,29,30]. Cefepime/zidebactam exhibits robust in vitro and translational activity against Enterobacterales, including many carbapenem-resistant phenotypes, and strong activity against *P. aeruginosa*, while its role against *A. baumannii* remains limited [10,12,14,31,32,33,34,35,36,37,38,39]. Cefepime/taniborbactam provides broad β-lactamase inhibition and retains substantial activity against OXA-48-, KPC-, VIM-, and most NDM-producing Enterobacterales, but lacks efficacy against IMP- and certain NDM-variant–producing isolates, reflecting inherent limitations in its spectrum [10,12,37,38,39,40,41,42,43,44,45,46,47,48].

Observed variations in susceptibility across geographic regions highlight the influence of local epidemiology, including the prevalence of specific β-lactamase variants, clonal expansion, and resistance determinants such as porin loss, PBP alterations, efflux pump overexpression [10,12,24,28,38,44,101,102,103,104]. These differences underscore the need for region-specific surveillance to guide empiric therapy and optimize clinical outcomes.

Their integration into clinical practice relies on robust in vitro susceptibility testing and standardized methodologies capable of accurately assessing activity against contemporary resistant isolates and emerging β-lactamase variants. Continuous surveillance is essential to detect and monitor resistance mechanisms, including PBP modifications, porin loss, efflux pump overexpression, and the emergence of novel KPC or NDM variants, that may compromise long-term efficacy. Anticipating this evolutionary trend will be critical to guide therapeutic decision-making and preserve the clinical utility of these combinations.

Cefepime-based combinations hold promise across a broad spectrum of serious infections, including cUTIs, bloodstream infections, hospital-acquired pneumonia, and other severe infections caused by extensively drug-resistant pathogens. Their optimal use may involve tailored dosing strategies guided by pharmacokinetic/pharmacodynamic principles, as well as rational combination approaches in the setting of complex resistance phenotypes.

Future research priorities include the establishment of clinical breakpoints, expansion of efficacy data in pneumonia, intra-abdominal infections, and bacteremia, and evaluation of combination regimens designed to overcome emerging resistance mechanisms. Real-world implementation will require coordinated antimicrobial stewardship efforts, routine susceptibility testing, and ongoing resistance surveillance to balance clinical efficacy with sustainability.

Overall, cefepime combined with late-generation BLIs exemplifies a strategic evolution in β-lactam therapy. By leveraging novel BLIs and β-lactam enhancers, these agents offer versatile and effective options to reduce carbapenem reliance while maintaining robust activity against multidrug-resistant Gram-negative pathogens, thereby strengthening the therapeutic armamentarium against an escalating global threat [10,105,106].

## Figures and Tables

**Table 1:** Novel cefepime-based combinations, approved or under clinical investigation, with activity against multidrug-resistant Gram-negative species.

Combination	β-Lactamase Inhibitor Characteristics	Year of FDA/EMA Approval	Clinical Trial	Targeted Species	Resistance Mechanisms
Cefepime/enmetazobactam	DBO β-lactamase inhibitor	2024 ^a^	NCT03687255 (Phase III, ALLIUM trial)	ESBL-producing Enterobacterales	Carbapenemase production, overexpression of ESBL enzymes, PBP-3 mutations, porin loss, and efflux pump overexpression.
Cefepime/zidebactam	DBO β-lactamase inhibitor with direct activity on PBP-2	na	NCT04979806 (Phase III, completed)	Enterobacterales, *P. aeruginosa*, *A. baumannii*	Multiple mutations in genes encoding MexAB-OprM and its regulators, as well as PBP-2 and PBP-3; porin loss; *bla*_PER-1_ overexpression, expression of KPC β-lactamases with 270-loop alterations
Cefepime/taniborbactam	Cyclic boronate β-lactamase inhibitor	na	NCT03840148 (Phase III, CERTAIN-1 trial) NCT06168734 (Phase III, withdrawn)	Enterobacterales, *P. aeruginosa*	IMP-like, NDM-9, NDM-30 or NDM-60 expression, alterations in PBP-3, porins loss, upregulation of efflux pumps

^a^ Approved by both the FDA and the EMA for the treatment of complicated urinary tract infections, including acute pyelonephritis, in adults. In the European Union, approval also includes hospital-acquired pneumonia, including ventilator-associated pneumonia, and bacteremia associated with these infections. Abbreviations: na, not available (approval is still pending); FDA, Food and Drug Administration; EMA, European Medicines Agency; PBP, penicillin binding protein; DBO, diazabicyclooctane β-lactamase inhibitor.

**Table 2:** Spectrum of activity of cefepime combined with novel β-lactamase inhibitors.

	Enterobacterales					*P. aeruginosa* (MDR/XDR)	*Acinetobacter* (MDR/XDR)	*S. maltophilia*
	ESBL	AmpC	KPC	mβL	OXA-48			
Cefepime/enmetazobactam								
Cefepime/taniborbactam								
Cefepime/zidebactam								

Green: antimicrobial activity, red: no antimicrobial activity, yellow: variable activity, restricted to certain β-lactamase types or variants and/or a limited subset of bacterial strains. Abbreviations: ESBL: extended-spectrum β-lactamase; AmpC: cephalosporinase, Ambler Class C β-lactamases; KPC: *Klebsiella pneumoniae* carbapenemase, mβL: metallo-β-lactamases, OXA-48: oxacillinase-48, Ambler Class D β-lactamases; MDR: multidrug resistant; XDR: extended drug resistant.

**Table 3:** In vitro activity of cefepime/enmetazobactam against collections of clinical Gram-negative isolates.

Reference	Country	Period of Isolates Collection	Species	Phenotype/Genotype	No.				Susceptibility		
						MIC50 [mg/L]	MIC90 [mg/L]	Range	EUCAST [≤4 mg/L]	FDA [≤8 mg/L]	Other Breakpoints
Morrissey [19]	USA, Europe	2014–2015	Enterobacterales	All	1696	0.06	0.25	0.015 to >64	97.3	98.1	na
			*E. coli*	ESBL	109	0.06	0.12	0.016 to 32	99.1	99.1	na
			*K. pneumoniae*	ESBL	102	0.12	1	0.03 to 8	98	100	na
			*K. pneumoniae*	KPC	45	16	>64	0.5 to >64	24.4	42.2	na
			*P. aeruginosa*	All	297	4	16	0.12 to >64	na	82.8	82.8 ^a^
Tselepis [20]	Wales (UK), India, Spain, Brazil, and Egypt	na	Enterobacterales	ESBL	107	≤0.06	0.125	na	na	na	98 ^b^
				KPC	117	32	64	na	na	na	na
				OXA-48-like	33	2	>64	na	na	na	33 ^b^
Vázquez-Ucha [10]	Spain	2018	Enterobacterales	CP	400	1	≥128	≤0.5 to >128	67.8	72.5	na
				CP, Non-ESBL	106	2	≥128	≤0.5 to >128	53.8	56.6	na
				CP, ESBL	294	1	32	≤0.5 to >128	72.8	78.2	na
				OXA-48like	304	1	16	na	na	na	74.2 ^c^
				KPC	44	64	≥128	na	na	na	40.9 ^c^
				mβL	56	64	≥128	na	na	na	3.6 ^c^
Belley [21]	Europe, USA	2016–2018	Enterobacterales	All	7168	0.03	0.25	≤0.008 to >64	na	98.8	98.3 ^c^
				ESBL	801	0.06	0.5	0.015 to 32	na	99.9	98.9 ^c^
			*E. coli*	ESBL	418	0.06	0.5	0.015 to 8	na	100	99 ^c^
			*K. pneumoniae*	ESBL	299	0.06	0.5	0.015 to 32	na	99.7	98.7 ^c^
Liu [22]	Taiwan	2018–2020	*P. aeruginosa*	CR	150	8	32	0.5 to >64	na	58	na
			*A. baumannii*	CR	255	32	64	1 to >128	na	na	na
Lee [23]	Taiwan	2017–2020	Enterobacterales	CnS	201	4	>64	≤0.03 to >64	na	na	84.6 ^c^
			*E. coli*	CnS	26	1	4	≤0.03 to 8	na	na	na
			*K. pneumoniae*	CnS	175	4	>64	≤0.03 to >64	na	na	na
Bonnin [24]	French	2021–2023	Enterobacterales	CnS	2212	na	na	na	na	na	na
				CP	2089	na	na	≤0.25 to >16	na	na	na
				OXA-48like	1000	≤0.25	2	4 to >16	98.8	na	na
				NDM	601	16	>16	4 to >16	0.9	na	na
				VIM	178	8	>16	1 to >16	60.6	na	na
				KPC	51	2	>16	≤0.25 to >16	63.3	na	na
				non-CP	123	2	>16	≤0.25 to >16	78.5	na	na
Morrissey [25]	Europe	2019–2021	Enterobacterales	All	2627	0.03	0.25	≤0.015 to >64	97.9	na	na
				ESBL	206	0.06	0.25	0.03 to 2	100	100	na
				AmpC	85	0.25	1	0.03 to 4	100	100	na
Bakthavatchalam [26]	India	2023–2024	Enterobacterales	All	625	≤0.12	≤0.12	≤0.12 to 128	98.7	100	na
			*E. coli*	ESBL/AmpC	321	≤0.12	≤0.12	≤0.12 to 4	100	100	na
			*K. pneumoniae*	ESBL/AmpC	200	≤0.12	≤0.12	≤0.12 to 2	100	100	na
			*K. pneumoniae*	OXA-48 like	50	4	128	4 to 128	16	40	na
			Other species	ESBL	54	0.25	≤0.12	≤0.12 to 2	100	100	na
			*P. aeruginosa*	All	100	2	8	0.25 to 8	na	100	na
Rezzoug [27]	France	2024–2025	Enterobacterales	CR, non-CP	151	2	>16	na	68.9	na	na
Dutkiewicz [28]	France	2024	Enterobacterales	KPC	28	4	16	≤0.06 to >16	54	na	na
				NDM	423	16	16	4 to 16	3	na	na
				VIM	119	8	16	0.5 to >16	30.2	na	na
				OXA-48	1334	≤0.06	2	≤0.06 to >16	94.1	na	na
				NDM+OXA-48	50	16	16	8 to >16	0	na	na
				OXA-23	9	≤0.06	0.12	na	100	na	na

Cefepime/enmetazobactam was tested using fixed enmetazobactam concentration of 8 mg/L. ^a^ CLSI 2019 cefepime susceptibility breakpoint (≤8 mg/L); ^b^ Cefepime EUCAST 2019 susceptibility breakpoint: S: ≤1 mg/L; ^c^ Cefepime CLSI 2019 susceptibility breakpoint: S: ≤2 mg/L. Abbreviations: na, non applicable; CR, carbapenem resistant; CP, carbapenemase producers; non-ESBL, ESBL-non-producing; mβL, metallo-β-lactamase, CnS, carbapenem-non-susceptible; non-CP, carbapenemase-non-producing.

**Table 4:** In vitro activity of cefepime/zidebactam against collections of clinical Gram-negative isolates.

Reference	Country	Period of Isolates Collection	Species	Phenotype/Genotype	No.				Susceptibility		
						MIC50 [mg/L]	MIC90 [mg/L]	Range	EUCAST ^a^	CLSI ^a^	PK/PD ^a^
Sader [14]	Worldwide	2015	Enterobacteriacae	All	5946	≤0.03	0.12	≤0.03 to 64	na	na	99.9
				CR	153	1	4	0.06 to 64	na	na	99.3
			*P. aeruginosa*	All	1291	1	4	0.06 to 32	99.5	99.5	na
			*Acinetobacter* spp.	All	639	16	32	0.06 to 64	na	44.3	na
Khan [31]	New York	2017	Enterobacterales	All	2560	≤0.03	0.06	≤0.03 to 4	100	na	na
			*P. aeruginosa*	All	271	2	8	0.25 to 256	98.5	98.5	na
			*A. baumannii*	All	46	4	16	≤0.03 to 16	na	84.8	na
Yang [32]	China	2018	*E. coli*	All	719	0.06	0.12	≤0.03 to 32	na	99	na
			*E. coli*	CR	33	0.12	8	≤0.03 to 32	na	81.8	na
			*K. pneumoniae*	All	788	0.12	2	≤0.03 to 32	na	96.3	na
			*K. pneumoniae*	CR	272	2	64	0.06 to 32	na	89.7	na
			*P. aeruginosa*	All	657	2	8	0.06 to 64	na	97.4	na
			*P. aeruginosa*	CR	171	4	8	1 to 64	na	93	na
Karlowsky [33]	Worldwide		Enterobacterales	CnS	1018	0.5	4	≤0.03 to >64	na	na	98.5
				mβL	191	0.5	4	na	na	na	94.8
				KPC	561	0.5	2	na	na	na	100
			*P. aeruginosa*	MDR	262	8	16	0.5 to >64	na	59.9	99.6
				mβL	94	8	16	na	na	na	100
			*S. maltophilia*	All	101	8	32	1 to 64	na	na	na
Mushtaq [34]	UK	2015–2016	Enterobacterales	KPC	116	0.25	1	0.03 to 2	100	100	na
				GES	10	0.5	4	0.12 to 4	100	80	na
				mβL	210	1	8	0.03 to 128	82.8	70.9	na
				OXA-48	250	0.12	1	0.03 to 2	100	100	na
				ESBL	307	0.25	1	0.03 to 8	99.7	98.4	na
				AmpC	418	0.25	0.5	0.03 to 2	100	100	na
			*P. aeruginosa*	mβL	81	8	8	2 to 16	93.8	93.8	na
				GES	15	2	4	1 to 4	100	100	na
				AmpC	71	4	8	0.5 to 16	98.6	98.6	na
				Efflux raised	188	4	8	0.03 to 32	92.5	92.5	na
				Efflux highly raised	85	8	16	0.25 to 32	84.1	84.1	na
			*A. baumannii*	OXA carb	183	8	32	0.06 to 128	na	53.5	na
				mβL	19	128	>128	32 to >128	na	0	na
			*S. maltophilia*	All	32	2	4	0.12 to 32	na	na	na
Sader [35]	Worldwide		Enterobacterales	All	17,524	0.03	0.25	≤0.008 to >64	99.8	99.7	na
				CR	681	1	2	≤0.008 to >64	96.3	91.9	na
				ESBL	2889	0.12	0.25	≤0.008 to 2	100	100	na
			*P. aeruginosa*	All	4808	1	4	≤0.008 to 64	99.2	99.2	na
				Mem non-S	1147	4	8	0.12 to 64	96.6	96.6	na
				Pip/Taz non-S	1122	4	8	0.5 to 64	96.5	96.5	na
				Caz/avi non-S	264	8	16	0.5 to 64	89	89	na
				Cft/taz non-S	286	4	8	na	90.9	90.9	na
			*A. baumannii*	All	1139	16	32	0.015 to >64	na	47.4	na
			*S. maltophilia*	All	636	4	16	0.5 to >64	na	na	na
Liu [22]	Taiwan	2018–2020	*P. aeruginosa*	CR	150	4	8	0.5 to >128	94.7	94.7	na
			*A. baumannii*	CR	255	32	64	1 to >128	na	na	na
Guo [36]	China	2019	Enterobacterales	All	2656	0.06	1	≤0.03 to >64	na	na	98.5
				CR	379	1	4	na	na	na	96
				NDM	117	0.25	16	na	na	na	89.7
				KPC	243	1	2	na	na	na	98.8
			*P. aeruginosa*	All	756	2	8	≤0.03 to >64	na	na	98.9
				CR	228	4	8	na	na	na	98.2
			*A. baumannii*	All	630	16	32	na	na	na	97.3
				CR	471	16	64	na	na	na	96.6
Bakthavatchalam [37]	India	2019–2021	*E. coli*	NDM	211	0.12	0.5	≤0.06 to 2	100	100	100
				NDM+OXA-48like	39	0.12	0.5	≤0.06 to 4	100	97.4	100
				OXA-48like	20	0.12	1	≤0.06 to 4	100	95	100
			*K. pneumoniae*	NDM	47	0.5	4	≤0.06 to 8	93.6	85.1	100
				NDM+OXA-48like	122	1	8	≤0.06 to 32	88.5	73.8	99.2
				OXA-48like	131	1	2	≤0.06 to 2	100	100	100
Liu [38]	China	na	Enterobacterales	NDM	204	1	32	0.06 to >64	61.3	52.9	na
Petillon [39]	France	na	Enterobacterales	3GC-R	200	0.12	0.25	≤0.06 to 0.5	100	100	na
				CR	414	0.25	2	≤0.06 to 16	88.9	94.9	na
				CP	292	0.12	0.5	≤0.06 to 16	96.9	98.3	na
Vázquez-Ucha [10]	Spain	2018	Enterobacterales	CP	400	≤0.5	1	≤0.5 to 8	na	99	na
				OXA-48	304	≤0.5	≤0.5	na	na	75.7	na
				KPC	44	≤0.5	1	na	na	100	na
				mβL	56	≤0.5	1	na	na	96.4	na
Dutkiewicz [28]	France	2024	Enterobacterales	KPC	28	0.25	1	≤0.06 to 1	100	na	na
				NDM	423	0.12	1	≤0.06 to 16	96	na	na
				VIM	119	0.12	0.5	≤0.06 to 2	100	na	na
				OXA-48	1334	≤0.06	0.25	≤0.06 to 8	99	na	na
				NDM+OXA-48	50	0.5	2	≤0.06 to >16	94	na	na
				OXA-23	9	≤0.06	0.12	na	100	na	na

Cefepime and zidebactam were tested at a ratio of 1:1. ^a^ Susceptibility data were interpreted using EUCAST (2024), CLSI (2024), and provisional PK/PD breakpoints as follows: EUCAST cefepime susceptibility breakpoint (v_14.0, 2024): Enterobacterales, ≤4 mg/L; *Pseudomonas*, ≤8 mg/L. CLSI cefepime susceptibility breakpoints (CLSI M100 ED34:2024): Enterobacterales, ≤2 mg/L; Pseudomonas, ≤8 mg/L; *Acinetobacter*, ≤8 mg/L. Provisional PK/PD susceptibility breakpoints: Enterobacterales, ≤8 mg/L; *Pseudomonas* ≤32 mg/L; *Acinetobacter* ≤64 mg/L. Abbreviation: na, non applicable; CR, carbapenem resistant; CP, carbapenemase producers; mβL, metallo-β-lactamase, CnS, carbapenem-non-susceptible; MDR, multi-drug-resistant; Mem, meropenem, Pip/Taz, piperacillin/tazobactam; Caz/avi, ceftazidime/avibactam; Cft/taz, ceftolozane/tazobactam; non-S, non-susceptible; 3GC-R, third generation cephalosporins resistant.

**Table 5:** In vitro activity of cefepime/taniborbactam against collections of clinical Gram-negative isolates.

Reference	Country	Period of Isolates Collection	Species	Phenotype/Genotype	No.				Susceptibility		
						MIC50 [mg/L]	MIC90 [mg/L]	Range [mg/L]	EUCAST ^a^	CLSI ^a^	Other Breakpoints
Bakthavatchalam [37]	India	2019–2021	*E. coli*	NDM	211	32	>64	0.5 to >64	5.2	4.7	12.3 ^b^
				NDM+OXA-48like	39	32	>64	1 to >64	7.7	5.1	10.3 ^b^
				OXA-48like	20	1	4	≤0.06 to 64	95	95	95 ^b^
			*K. pneumoniae*	NDM	47	2	32	≤0.06 to >64	72.3	57.4	74.5 ^b^
				NDM+OXA-48like	122	32	>64	0.5 to >64	13.9	9.8	18.9 ^b^
				OXA-48like	131	4	4	≤0.06 to 8	96.1	80.9	100 ^b^
Liu [38]	China	na	Enterobacterales	NDM	204	2	32	0.06 to >64	67.1	51	na
Vázquez-Ucha [10]	Spain	2018	Enterobacterales	CPE	400	≤0.5	2	≤0.5 to ≥128	na	90	na
				OXA-48	304	≤0.5	2	na	na	93.1	na
				KPC	44	1	4	na	na	84.1	na
				mβL	56	1	8	na	na	75	na
Wang [41]	China	2017–2019	Enterobacterales	ESBL	56	0.03	0.12	≤0.008 to 0.5	100	100	na
				AmpC	61	0.06	0.25	0.016 to2	100	100	na
				ESBL+AmpC	32	0.12	1	0.016 to 4	100	96.9	na
				KPC	66	2	8	0.03 to 64	89.4	74.2	na
				NDM	87	16	64	0.03 to 128	41.4	33.3	na
			*P. aeruginosa*	CR	22	8	32	4 to 64	68.2	68.2	na
				CS	28	4	8	0.5 to 8	100	100	na
Meletiadis [42]	Greece	2019–2020	*K. pneumoniae*	mβL	100	2	16	0.5 to 128	78	61	na
			*P. aeruginosa*	mβL	100	16	32	1 to 64	46	46	na
Kloezen [43]	na	na	*E. coli*	ESBL	42	na	0.5	0.06 to 2	100	na	na
			*K. pneumoniae*	ESBL	39	na	1	0.06 to 4	100	na	na
			*P. aeruginosa*	ESBL	28	na	16	2 to 64	64	na	na
Karlowsky [44]	Worldwide	2018–2020	Enterobacterales	All	13,731	0.06	0.25	≤0.008 to >16	na	na	99.7 ^c^
				ESBL	4335	0.12	1	≤0.008 to >16	na	na	99.1 ^c^
				CR	637	1	8	0.016 to >16	na	na	94.5 ^c^
				CP	633	1	16	0.016 to >16	na	na	94.6 ^c^
				MβL	235	1	>16	0.03 to >16	na	na	86.4 ^c^
				KPC	230	0.5	4	0.016 to 8	na	na	100 ^c^
				OXA-48	168	2	4	0.03 to >16	na	na	98.8 ^c^
			*P. aeruginosa*	All	4619	2	8	≤0.06 to >32	na	na	97.4 ^c^
				CR	1415	8	16	0.25 to >32	na	na	92.4 ^c^
				CP	249	8	>32	1 to >32	na	na	71.5 ^c^
				mβL	209	8	>32	1 to >32	na	na	66.5 ^c^
Hernández-García [45]	Spain	2020	Enterobacterales	CP	247	0.5	4	≤0.06 to 32	na	na	97.6 ^b^
				KPC	35	2	8	na	na	na	94.3 ^b^
				OXA-48	15	4	8	na	na	na	100 ^b^
				mβL	16	2	16	na	na	na	87.5 ^b^
			*P. aeruginosa*	All	163	8	32	≤0.06 to 32	na	na	67.5 ^b^
				CR	122	8	32	na	na	na	63.9 ^b^
				GES	30	8	8	na	na	na	93.8 ^b^
				VIM	49	8	>32	na	na	na	61.2 ^b^
Jacobs [46]	USA	2012–2026	*K. pneumoniae*	CnS/CP	200	0.25	2	≤0.06 to 16	na	na	99.5 ^b^/100 ^c^
			*P. aeruginosa*	All	197	4	16	0.5 to >128	na	na	82.7 ^b^/92.4 ^c^
				CnS	108	8	32	1 to >128	na	na	69.4 ^b^/86.1 ^c^
				KPC	20	16	128	4 to >128	na	na	35 ^b^/60 ^c^
Santerre Henriksen [47]	Europe	2020	Enterobacterales	All	1909	na	0.5	na	na	98.7	na
				CR	148	na	4	0.12 to 16	na	93.2	na
Wang [48]	Taiwan	2020–2022	*E. coli*	CnS	29	0.5	>32	≤0.06 to >32	na	na	72.4 ^b^
			*K. pneumoniae*	CnS	138	2	8	≤0.06 to >32	na	na	95.7 ^b^
			*P. aeruginosa*	CnS	85	8	16	1 to >32	na	na	70.6 ^b^
			*A. baumannii*	CnS	135	>32	>32	8 to >32	na	na	5.2 ^b^
Dutkiewicz [28]	France	2024	Enterobacterales	KPC	28	0.5	2	≤0.06 to 4	100	na	na
				NDM	423	1	16	0.12 to >16	76	na	na
				VIM	119	0.12	1	≤0.06 to 8	98.3	na	na
				OXA-48	1334	≤0.06	1	≤0.06 to >16	97.6	na	na
				NDM+OXA-48	50	8	>16	0.5 to >16	26	na	na
				OXA-23	9	0.12	0.25	na	100	na	na

For susceptibility testing purpose, the concentration of taniborbactam was fixed at 4 mg/L. ^a^ Susceptibility data were interpreted using EUCAST (2024), CLSI (2024) as follows: EUCAST cefepime susceptibility breakpoint (v_14.0, 2024): Enterobacterales, ≤4 mg/L; *Pseudomonas*, ≤8 mg/L. CLSI cefepime susceptibility breakpoints (CLSI M100 ED34:2024): Enterobacterales, ≤2 mg/L; *Pseudomonas*, ≤8 mg/L; *Acinetobacter*, ≤8 mg/L. ^b^ Provisional susceptibility breakpoints ≤8 mg/L. ^c^ Provisional susceptibility breakpoints ≤16 mg/L. Abbreviation: na, non applicable; CR, carbapenem-resistant; CP, carbapenemase producers; CS, carbapenem-susceptible; mβL, metallo-β-lactamase; CnS, carbapenem-non-susceptible.

## Data Availability

No new data were created or analyzed in this study. Data sharing is not applicable to this article.
